# High Expression of Succinate Dehydrogenase Subunit A Which Is Regulated by Histone Acetylation, Acts as a Good Prognostic Factor of Multiple Myeloma Patients

**DOI:** 10.3389/fonc.2020.563666

**Published:** 2020-09-10

**Authors:** Yifeng Sun, Zhao Xu, Jifeng Jiang, Tianhong Xu, Jiadai Xu, Peng Liu

**Affiliations:** Department of Hematology, Zhongshan Hospital, Fudan University, Shanghai, China

**Keywords:** multiple myeloma, succinate dehydrogenase subunit A, proliferation and invasion, synergistic effect, histone acetylation

## Abstract

Most patients with multiple myeloma (MM) will eventually relapse and current treatments have limited effect. Herein, we demonstrate that succinate dehydrogenase subunit A (SDHA) was low expressed in MM patients, and patients with SDHA relatively high expression had long overall survival and progression-free survival. Furthermore, SDHA high expression inhibited proliferation and invasion in MM cell lines and enhanced the anti-tumor and synergistic effect of chemotherapeutics. More importantly, chidamide was proved effective in MM by targeting SDHA, and expression of SDHA was increased by chidamide through acetylating H3K27 site of SDHA. Collectively, high expression of SDHA, which was regulated by histone acetylation and targeted by chidamide, might become a good prognostic factor of MM patients.

## Introduction

Multiple myeloma (MM) is a common hematological malignancy characterized by proliferation of monoclonal malignant plasma cells and production of monoclonal immunoglobulin (1). MM is the second most frequent hematological cancer, accounting for about 1% of all kinds of cancer and 13% of hematological tumors (2). More than 100,000 patients die of MM every year worldwide (3). Typical clinical manifestations include osteolytic lesions with bone pain, monoclonal protein in serum, anemia of unknown origin, hypercalcemia or renal insufficiency (4). Currently in clinical, common staging of MM includes three main staging systems. Durie/Salmon (DS) staging system is depended on concentration of hemoglobin and serum calcium, the number of osteolytic lesions and production of serum immunoglobulin and urine Bence Jones protein (5). International Staging System (ISS) is determined by quantity of serum β2-microglobulin and albumin (6). In 2015, on the basis of ISS staging system, International Myeloma Working Group (IMWG) added molecular genetic abnormality and level of lactate dehydrogenase (LDH) in the system, which was named as Revised International Staging System (R-ISS) (7). These staging systems were regarded as common prognostic criteria of MM patients.

Multiple myeloma has very complicated pathogenesis and high tumor heterogeneity (8). This heterogeneity not only reflects in complicated genetic abnormalities, but also epigenetic abnormalities (9). As a new histone deacetylase inhibitor (HDACi), chidamide has been used in malignancy treatment (10) by targeting histone deacetylase (HDAC) type I, which is closely related to tumorgenesis and progression (11). To date, chidamide has been approved in China for oral treatment of recurrent or refractory peripheral T-cell lymphoma (PTCL) (12). In our previous study, we found chidamide could inhibit proliferation and inhibition of MM cells (13).

Generally speaking, the main mechanism of chidamide is acetylating histone of tumor suppressor genes (14). In our previous study, we screened succinate dehydrogenase subunit A (SDHA) as the most important target of chidamide in MM cells by RNA sequencing. We have demonstrated that knocking down SDHA would make chidamide invalid. In this study, combined RNA and chromatin immunoprecipitation (ChIP) sequencing analysis, we linked the anti-tumor effect of chidamide to enhanced expression of SDHA. Succinate dehydrogenase (SDH) encodes an essential enzyme in oxygen metabolism and SDHA is the most important catalytic subunit of SDH (15). It is reported that deficiency of SDHA blocked tricarboxylic acid (TCA) cycle. Too much succinate accumulated leads to inhibition of HIFA degradation, and then production of reactive oxygen species (ROS) increased (16). High oxidative stress will drive tumor cells to be proliferative and invasive (17). In a word, deficiency of SDHA may lead to tumorigenesis in some cancers and SDHA can be regarded as tumor suppressor gene in these cancers.

This study aims to explore the relationship between expression of SDHA and prognosis of MM patients. In the present study, we found patients with high SDHA expression had better prognosis. Based on the expression of SDHA, R-ISS and percentage of plasma cells in bone marrow of patients, we raised a new prognosis prediction model of MM. *In vitro*, we verified SDHA overexpression led MM cells to be low proliferative and invasive and high synergistic effect under chidamide and bortezomib or lenalidomide treatment, which indicated that high expression of SDHA was a good prognostic factor of MM.

## Materials and Methods

### Patients

The histological diagnosis was established according to IMWG 2014 criteria (3). The study was approved by Shanghai Zhongshan Hospital Review Board and informed consent was obtained from patients in accordance with the Declaration of Helsinki. Bone marrow samples were obtained from MM patients. Bone marrow-derived mononuclear cells (BMMCs) were isolated from the bone marrow samples by Ficoll-isopaque centrifugation.

### Cells and Reagents

Bone marrow-derived mononuclear cells and MM cell lines were cultured in RPMI-1640 medium with 10% heat-inactivated fetal bovine serum (FBS) in a humidified atmosphere of 95% air and 5% CO_2_ at 37°C. MM cell lines H929, OPM2 and U266 were purchased from American Type Culture Collection (ATCC, Manassas, VA, United States). Chidamide was purchased from Selleck Chemicals (Houston, TX, United States).

### RNA Extraction

Total RNA was extracted from BMMCs. Then the integrity of the total RNA was determined by 2100 Bioanalyzer (Agilent Technologies, Santa Clara, CA, United States) and quantified by NanoDrop (Thermo Scientific, United States). About 1 μg high-quality RNA was used to construct sequencing library.

### cDNA Library Construction and RNA Sequencing

About 1 μg total RNA was used, and it was fragmented into small pieces using divalent cations under elevated temperature after purification of the remaining RNA. The cleaved RNA fragments were copied into first strand cDNA using reverse transcriptase and random primers, followed by second strand cDNA synthesis. Amplified target fragments were cleaned up by AMPure XP Beads (Beckmen, United States) to create the final cDNA library. RNA sequencing was performed by Illumina systems.

### ChIP Sequencing

Cells were washed by phosphate buffer saline (PBS) and then crosslinked with 1% formaldehyde at room temperature for 10 min. Crosslinking was stopped by addition of glycine to a final concentration of 0.15 M at room temperature for 5 min.

For each sample, 20 million fixed cells were lysed to prepare nuclear extracts. After chromatin shearing by sonication, lysates were incubated overnight at 4°C with protein A Dynabeads coupled with 5 μg antibody. After immunoprecipitation, beads were recovered using a magnet and washed. DNA was eluted, crosslinks reverted at 65°C for 4 h and then purified with QIAGEN Kit. DNA was quantitated using the Qubit^®^ dsDNA HS assay and a Qubit^®^ 2.0 Fluorimeter (Invitrogen). For ChIP-Seq, 5 ng of purified ChIP DNA were used to generate the sequencing library using a NEB kit and sequenced with the Illumina HiSeq X Ten. The normalization for reads count of the ChIP-Seq was referred to Reads Per Kilobase per Million mapped reads (RPKM).

### Western Blot

Western blot was performed as described previously (13). Antibodies against SDHA and HIFα were from Abcam (Cambridge, MA, United States). Actin (Cell Signaling technology, Beverly, MA, United States) was used to ensure equivalent protein loading. Statistical significance of the bar graph relative to the western blot presented by Image-pro plus (IPP) 6.0.

### Clinical Prognosis Analysis

105 BMMC samples were randomly selected to extract RNA with high quality at Zhongshan Hospital. Real-time RT-PCR was used to determine the expression of SDHA in these patients. MM patients were followed up by telephone or outpatient. The median follow-up time of 105 patients was 28 months, and the final update time of clinical follow-up data was July 2019.

We collected clinical and laboratory data of these 105 patients, including age, sex, diagnosis, DS staging, R-ISS staging, concentration of M-protein, abnormal serum free light chain existed or not, ratio of serum free light chain (κ/λ), lactate dehydrogenase (LDH), serum creatinine (SCr), percentage of plasma cells in bone marrow, abnormal results of fluorescence *in situ* hybridization (FISH), and Eastern Cooperative Oncology Group (ECOG) scoring.

The consecutive variables were analyzed by *t*-test. Categorial outcomes were compared by Chi-square test. The association between SDHA and outcomes was shown via Kaplan-Meier (K-M) curves. Then, multivariate stepwise Cox regression was performed and the model with lowest Akaike Information Criterion (AIC) was selected as the best to describe the outcomes of MM patients. Nomogram based on the Cox regression was constructed. Calibration curve was to evaluate the agreement between observed outcomes and predictions in our nomogram model. Besides, decision curve analysis (DCA) and concordance index (C-index) were performed to evaluate the discrimination of our model. All statistical tests were two-sided, and the analysis was made by SPSS 21 software and R software, version 3.6.0. *P* < 0.05 was regarded as statistical significance.

### Detection of Genes

Gene expression was analyzed by real-time RT-PCR using 7500HT Fast Real-time PCR system (Applied Biosystems, Foster City, CA, United States). Primers used were listed as below.

SDHA: Forward, 5′-TCGCACTGTGCATAGAGGAC-3′ and Reverse, 5′-ATGCCTGTAGGGTGGAACTG-3′.

ITGA7: Forward, 5′-ATCAAGATTTGGCAGGATCG-3′ and Reverse, 5′-ACACAGGGTGAATGGGAGAG-3′.

MEIS3: Forward, 5′-CTCACACCTGCCTCTGGTTC-3′ and Re-verse, 5′-GCAGAGGTGAAGGCAGAAGT-3′.

FCER2: Forward, 5′-ACACATCTCCCGCTCCTCTA-3′ and Reverse, 5′-CACCTGAGCTGGGGATACTC-3′.

MRPL30: Forward, 5′-ACGGTGGCTCACCTGTAA-TC-3′ and Reverse, 5′-TACAGTGGCACCATCT-CAGC-3′.

GAPDH: Forward, 5′-GAAGGTGAAGG-TCGGAGTC-3′ and Reverse, 5′-GAAGATGGTGA-TGGGATTTC-3.

Relative expressions were calculated by the method of ΔΔCT.

### Cell Transfection

Lenti-virus with SDHA or control vector was synthesized by Genomeditech (Shanghai, China). H929 and U266 cells were infected by lenti-virus with cell-virus ratio of 1:100. The transfected clones were detected after transfection for 72 h.

### Cell Proliferation Assay

Cells were seeded at a density of 2 × 10^5^ cells per well in 6 well plates and incubated at 37°C. Cell counts were calculated after 24 and 48 h, respectively.

### Cell Invasion Assay

Cell invasion was tested by Matrigel Invasion Chamber (BD Pharmingen, Franklin Lakes, NJ, United States), which was composed of the upper and lower compartment separated by membranes with matrigel and pores (8 μm pore size). About 6 × 10^4^ cells were incubated with RPMI-1640 (FBS-free, 200 μl) for 24 h and then added into the upper compartment, while RPMI-1640 with 10% FBS (500 μl) was added to the lower compartment. Cells were cultured for 24 h, and then invasive ability of cells was tested as described previously (18). The membrane was stained by Wright-Giemsa staining and the invading cells were observed.

### CCK8 Assay

Cells were seeded at a density of 5 × 10^5^ cells per well in 96 well plates and cultured at 37°C. 24 h later, 0.1 mg CCK8 was added to each well. Absorbance was measured at 490 nm by spectrophotometry.

### Synergistic Analysis

Combination index (CI) was calculated by formula (CI = D_A_/ICX_A_ + D_B_/ICX_B_ + D_A_ × D_B_/ICX_A_ × ICX_B_, A and B stood for a drug, respectively) (19). This method allows quantitative determination of drug interactions, where CI < 1, CI = 1, and CI > 1 indicate synergism, additive effect, and antagonism, respectively.

### Detection of ROS Accumulation

Mitochondrial ROS production was measured as described (20). CM-H2DCFDA (5 mM) was added 30 min before collecting cells. ROS production was detected by flow cytometry.

### Statistical Analysis of Experiments

Experimental results obtained from three separate experiments and determined by *t*-test to compare variance. *P* < 0.05 was considered statistically significant.

## Results

### SDHA Was a Good Prognostic Factor of Multiple Myeloma Patients

To test if current therapeutic drugs of MM were able to affect expression of SDHA, we used 10.67 μM bortezomib, 2.48 mM lenalidomide, 6 μM chidamide and isometric DMSO to treat H929 cells for 24 h, respectively. Western blot analysis showed that only chidamide increased the expression of SDHA, whereas expression of SDHA stayed at the same level in bortezomib and lenalidomide treated cells compared with control (Supplementary Figure S1A). BMMCs of 4 MM patients were randomly chosen to analyze the changes of SDHA before and after conventional chemotherapy treatment. The results of real-time RT-PCR reflected that there was no difference between the expression of SDHA in newly diagnosed patients and patients after treatment (Supplementary Figure S1B). To determine the effect of regular HDACi on regulating SDHA, we also tested the expression of SDHA in MM cell lines by real-time RT-PCR after treating with three kinds of HDACi, including valproic acid (VPA), vorinostat (SAHA) and chidamide. Results showed that only chidamide could increase the expression of SDHA (Supplementary Figure S2, *p* = 0.0064 for H929 cells, *p* = 0.0058 for OPM2 cells and *p* = 0.0183 for U266 cells).

In order to explore whether there was a relationship between expression of SDHA and prognosis of MM patients, real-time RT-PCR was used to determine the expression of SDHA in 105 MM patients. Based on trisection points of expression of SDHA, these patients were divided into three groups, including SDHA low expression group, SDHA middle expression group and SDHA high expression group. Table 1 showed the baseline characteristics of these 105 MM patients.

**TABLE 1:** Basic characteristics of 105 MM patients.

Variable	Low SDHA expression	Middle SDHA expression	High SDHA expression	*P*-value
**Categorical variable**				
Sex (male)	22 (62.9%)	24 (68.6%)	20 (57.1%)	0.613
DS staging				0.192
IA	5 (14.3%)	3 (8.6%)	6 (17.1%)	
IB	0 (0%)	0 (0%)	1 (2.9%)	
IIA	4 (11.4%)	0 (0%)	6 (17.1%)	
IIB	2 (5.7%)	2 (5.7%)	1 (2.9%)	
IIIA	20 (57.1%)	24 (68.6%)	13 (37.1%)	
IIIB	4 (11.4%)	6 (17.1%)	8 (22.9%)	
R-ISS staging				0.519
I	6 (17.1%)	5 (14.3%)	9 (25.7%)	
II	19 (54.3%)	24 (68.6%)	19 (54.3%)	
III	10 (28.6%)	6 (17.1%)	7 (20.0%)	
Abnormal free light chain exists	23 (65.7%)	28 (80.0%)	26 (74.3%)	0.396
Abnormal FISH exists	28 (80.0%)	31 (88.6%)	29 (82.9%)	0.612
Score of ECOG				0.192
1	6 (17.1%)	5 (14.3%)	5 (14.3%)	
2	10 (28.6%)	16 (45.7%)	17 (48.6%)	
3	14 (40.0%)	12 (34.3%)	11 (31.4%)	
4	5 (14.3%)	2 (5.7%)	2 (5.7%)	
**Continuous variable**				
Age	61.2	63.7	64	0.227
Concentration of M-protein (g/L)	17.4	23.9	16.6	0.272
Ratio of free light chain	80.5	37.1	43.7	0.441
LDH (IU/L).	231.9	220.5	193.7	0.419
Scr (μmol/L)	141.9	124	113.5	0.664
Percentage of plasma cells in bone marrow	27.1	30.2	18.7	0.034

**FIGURE 1:**
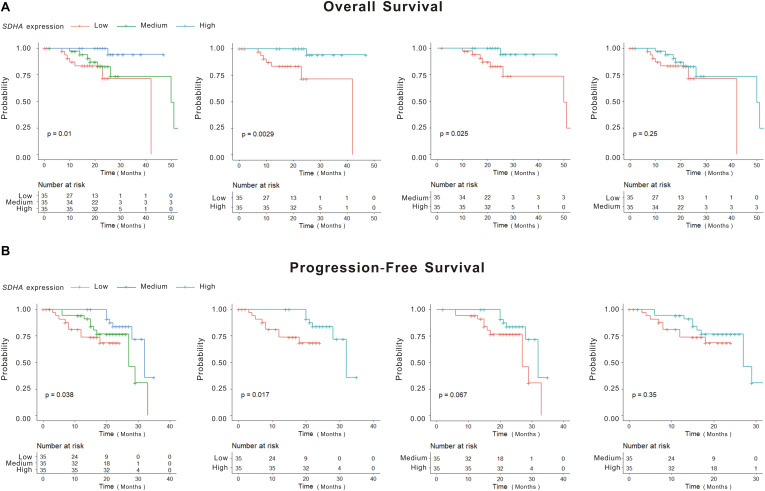
SDHA overexpression indicated longer OS and PFS in MM patients. **(A)** Kaplan-Meier (K-M) curves of overall survival of high *SDHA* expression group, medium *SDHA* expression group and low *SDHA* expression group. **(B)** Kaplan-Meier (K-M) curves of progression-free survival of high *SDHA* expression group, medium *SDHA* expression group and low *SDHA* expression group.

We analyzed the differences of overall survival (OS) and progression-free survival (PFS) among SDHA low expression group, SDHA middle expression group and SDHA high expression group patients. As shown in Figure 1, there were significant differences of OS and PFS among the three groups (*p* = 0.01 and *p* = 0.038, respectively). OS and PFS were significantly longer in SDHA high expression group than that in SDHA low expression group (*p* = 0.0029 and *p* = 0.017, respectively). OS in SDHA high expression group was significantly higher than that in middle SDHA expression group (*p* = 0.025). In addition, although there was no significant difference between OS in SDHA middle and low expression group and no significant difference between PFS in SDHA high and middle expression group and between SDHA middle and low expression group, SDHA high expression tended to be related to longer OS and PFS in MM patients.

We included expression of SDHA and all the variables shown in Table 1 into Cox multivariate regression analysis. To quantify the independent influence of variables, the multivariate stepwise Cox regression model with the minimum AIC was performed as in Supplementary Table S1. Hazard ratios (HRs) and *P*-values indicated that SDHA high expression (*P* = 0.0235) and percentage of plasma cells in bone marrow (*P* = 0.0201) were independently related to OS of MM patients. MM patients with high SDHA expression had significantly longer OS (*P* = 0.0235, HR = 0.374, 95% CI = [0.009, 0.713]).

**FIGURE 2:**
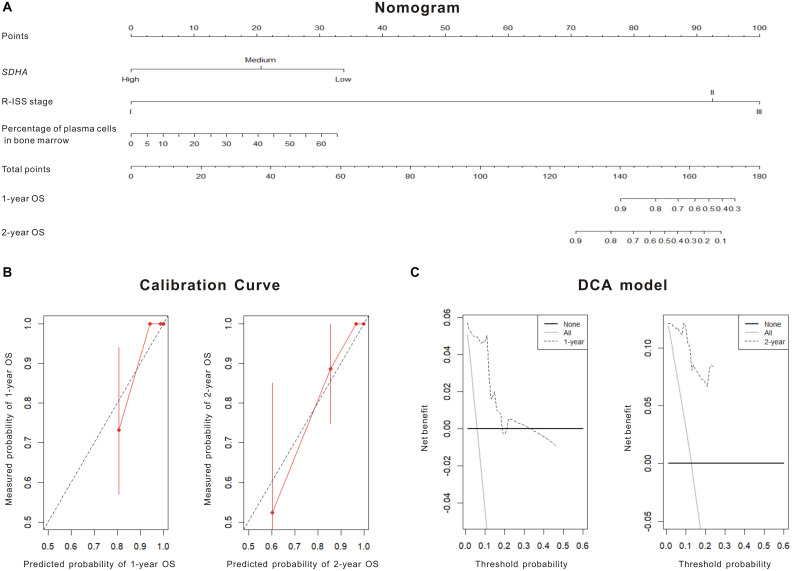
SDHA overexpression was involved in the prognosis model of MM patients. **(A)** Nomogram diagram shows three variables of the best prognosis prediction model in Cox regression, including expression of *SDHA*, R-ISS stage and percentage of plasma cells in bone marrow. The integral weight of each variable is shown in the figure. This diagram also predicted the relationship between this model and 1-year and 2-year survival rates of MM patients. **(B)** The calibration chart of patients’ prognosis model was based on Cox regression. The calibration curve was plotted to evaluate the agreement of nomogram. Calibration curve reflects the agreement between observed outcomes and predictions. A calibration plot has the predicted probabilities on the *x*-axis, and the mean observed outcome on the *y*-axis, and a perfect calibration should lie on or around a 45° line of the plot. Red solid line was the survival prediction of MM patients, while black virtual line was the actual survival rate of MM patients. **(C)** Decision curve analysis (DCA) of the 1 and 2-year overall survival. DCA was also used to evaluate the discrimination of the model and recent years have seen an explosion of interest in and practical use of decision curve analysis. The horizontal solid line referred to “none patient was dead” in 1- or 2-year follow-up and the thin line referred to “all patients were dead” in 1- or 2-year follow-up. The dotted line referred to the prediction of our 1- or 2-year model. All = assume all patients survive, None = assume none patient survives.

Although K-M curves were drawn to show high SDHA expression was correlated with better outcomes of MM patients, the specific survival probability of a MM patient could not be quantified even if we had all clinical profiles of this patient. The nomogram was constructed to solve this problem. Firstly, through stepwise, the model with the smallest AIC was obtained as the best survival prediction model. Then, the nomogram graph (Figure 2A) was generated to predict 1- and 2-year OS based on the statistically significant variables in the multivariate stepwise Cox regression model. The C-index of the nomogram was 0.871 (95% CI, 0.808–0.935) indicating a good discrimination of the nomogram. We could match the score of each factor in the nomogram and added them together to get the total points in order to predict the prognosis if patients. Fitting degree between the prediction model and the actual situation was shown by the calibration diagram (Figure 2B). Calibration diagram showed that this model had a high coincidence with the actual situation in predicting the prognosis of patients. DCA showed curves of this model were far from the two extreme curves demonstrating a very good net benefit (Figure 2C).

### Histone Acetylation of SDHA Was Upregulated by Chidamide in MM Cells

As shown in Supplementary Figure S3, RNA sequencing was performed previously in order to find out the target gene of chidamide in MM cells (13). As shown in Supplementary Figure S3, RNA sequencing was performed previously. Three newly diagnosed MM patients were involved in this study. We used bone marrow samples of these patients and BMMCs were isolated by Ficoll-isopaque centrifugation. Their BMMCs were each cultured as two groups, chidamide-treated group (CHI) and DMSO-treated group (CON). To investigate the target molecules of chidamide in MM cells, we performed RNA sequencing analysis to screen DEGs of CHI cells compared with CON cells. As shown in Supplementary Figure S3C, we set fold change >2, padj < 0.05 and false discovery rate (FDR) < 0.05 as thresholds to define DEGs. The detailed lists of the top 20 DEGs were attached in Supplementary Table S2 and the differential expression of DEGs were shown in Supplementary Figure S3D. Among them, the up-regulated genes mainly focus on cell adhesion, invasion, and angiogenesis regulators. By contrast, the down-regulated genes encoded ribosome protein or active monocytes and lymphocytes. The screening steps were also shown in Supplementary Figure S3 and SDHA was chosen as the probable target of chidamide in MM cells finally. To further verify signaling pathways and functional enrichment for each set of DEGs activated by chidamide, gene ontology (GO) and KEGG analysis was performed. The top 10 significantly enriched GO terms were showed in Supplementary Table S3. Results shown that endothelial cell differentiation, transforming growth factor beta-activated receptor activity and many metabolism pathways were involved in the chidamide-activated GO and KEGG pathways. Interestingly, TCA cycle regulation was ranked at the top of KEGG analysis. Finally, SDHA was chosen as the probable target of chidamide in MM cells.

**FIGURE 3:**
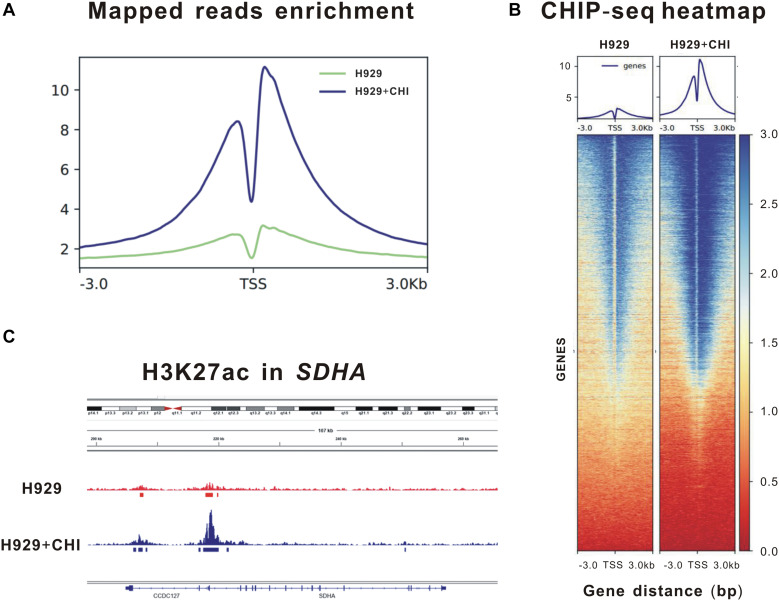
Enrichment of H3K27 acetylation of *SDHA* was raised by chidamide in MM cells. MM cell line H929 treated by chidamide or DMSO for 24 h and then ChIP sequencing was performed. **(A)** Enrichment of mapped reads of ChIP sequencing. The peak of enrichment of H3K27 acetylation of cells treated by chidamide was much higher than cells treated by DMSO. **(B)** The heat map of CHIP sequencing signal enrichment reflects the enrichment of all genes in the upstream and downstream 3 kb of transcription starting sites. The signal enrichment of H3K27 acetylation sites in chidamide-treated H929 cells was significantly higher than that in the control H929 cells. **(C)** The signal enrichment of H3K27 acetylation sites of *SDHA* gene in chidamide-treated H929 cells was significantly higher than that in the control H929 cells.

Theoretically speaking, as an epigenetic regulator, chidamide increases the expression of tumor suppressor genes by acetylating histone of them. Thus, to further clarify the epigenetic changes in histone of SDHA, we performed ChIP sequencing of MM cell line H929 treated by chidamide or DMSO for 24 h. Figure 3A showed enrichment of mapped reads and the peak of enrichment of H3K27 acetylation of cells treated by chidamide was much higher than cells treated by DMSO. ChIP sequencing heatmap of signal enrichment reflected that signal enrichment in H3K27 acetylation site of H929 cells treated with chidamide was higher than that treated with DMSO (Figure 3B). Then we checked the information of signal enrichment of H3K27 acetylation site in SDHA. As expected, signal enrichment of H3K27 acetylation site in SDHA of H929 cells treated with chidamide was much more than that treated with DMSO (Figure 3C). Thus it was reasonable to believe that SDHA mediated the effect of chidamide in MM cells.

### Upregulated Expression of SDHA Suppressed Proliferation of MM Cells and Enhanced Synergistic Effect of Chidamide and Chemotherapeutics

Since SDHA high expression was a good prognosis marker mentioned above, we constructed SDHA overexpression H929 and U266 cells by lenti-virus transfection to clarify the effect of SDHA high expression in MM cell lines. Western blot was used to determine the expression of SDHA after SDHA over-expression (OE), treatment with chidamide (CHI) and both (OE + CHI) in Supplementary Figure S4.

**FIGURE 4:**
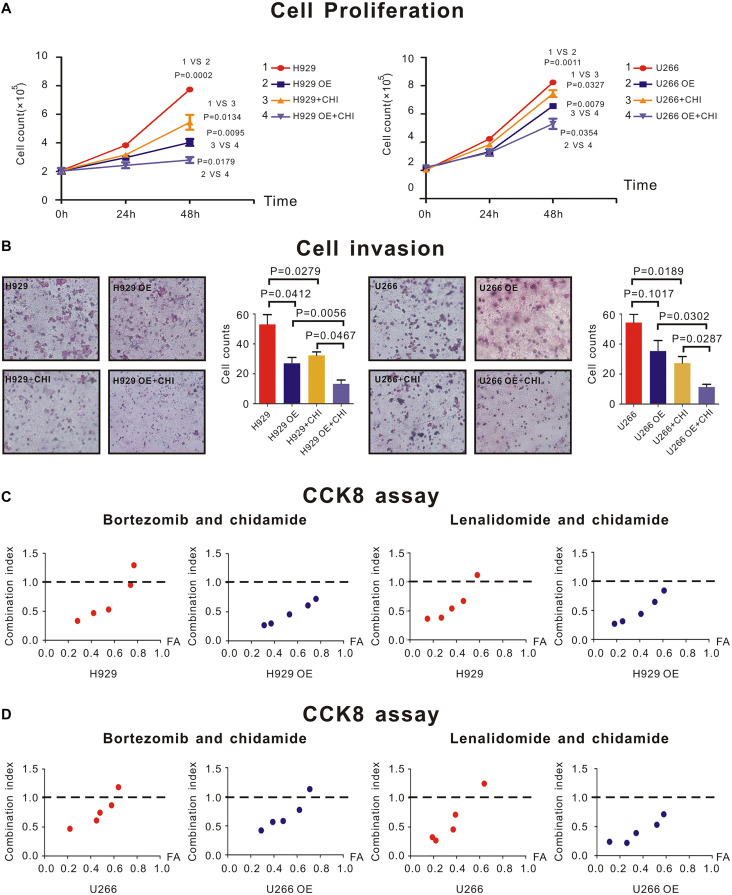
*SDHA* overexpression inhibited proliferation of MM cells and enhanced synergistic effect between chidamide and bortezomib or lenalidomide. **(A)** H929 and U266 cells were cultured with 6 μM chidamide or isometric DMSO for 48 h. *SDHA* overexpression (OE) cells had much lower proliferation rate than nc cells (1 vs. 2). Cells treated with chidamide proliferated much slower than cells treated by DMSO (1 vs. 3). Chidamide decreased proliferative ability of MM cells more significant in *SDHA* OE cells than nc cells (2 vs. 4). H929 (U266) stands for nc H929 (U266) cells. **(B)** H929 and U266 cells were cultured with 6 μM chidamide or isometric DMSO for 24 h. The number of cells which migrated through the transwell matrigel membrane reflected the relative ability of MM cells. *SDHA* overexpression (OE) cells had much lower invasive ability than nc cells. Cells treated with chidamide had much lower invasive ability than cells treated by DMSO. Chidamide decreased invasive ability of MM cells more significant in *SDHA* OE cells than nc cells. **(C)** CCK-8 kit was used to determine synergistic effect between two drugs. Left two panels showed combination index (CI) between bortezomib and chidamide at different inhibition rate (FA) in H929 cells. Compared with nc cells, CI was lower in *SDHA* OE cells at the same FA level. Right two panels showed combination index (CI) between lenalidomide and chidamide at different inhibition rate (FA) in H929 cells. Compared with nc cells, CI was lower in *SDHA* OE cells at the same FA level. **(D)** CCK-8 kit was used to determine synergistic effect between two drugs. Left two panels showed combination index (CI) between bortezomib and chidamide at different inhibition rate (FA) in U266 cells. Compared with nc cells, CI was lower in *SDHA* OE cells at the same FA level. Right two panels showed combination index (CI) between lenalidomide and chidamide at different inhibition rate (FA) in U266 cells. Compared with nc cells, CI was lower in *SDHA* OE cells at the same FA level. H929 (U266) represented nc H929 (U266) cells; H929 (U266) OE represented *SDHA* overexpression H929 (U266) cells. H929 (U266) + CHI represented nc H929 (U266) cells treated by 6 μM chidamide; H929 (U266) OE + CHI represented *SDHA* overexpression H929 (U266) cells treated by 6 μM chidamide.

H929 and U266 cells were cultured with an original concentration of 2 × 10^5^/L to detect proliferation of MM cells. Compared with normal control (nc), SDHA overexpression could inhibit cell proliferation in H929 (Figure 4A, left panel, 1 vs. 2, *P* = 0.0002) and U266 (Figure 4A, right panel, 1 vs. 2, *P* = 0.0011) cell lines. The proliferation rate of H929 (Figure 4A, left panel, 1 vs. 3, *P* = 0.0001) and U266 (Figure 3A, right panel, 1 vs. 3, *P* = 0.0052) could be inhibited by simply adding chidamide. When SDHA was overexpressed, this inhibition effect of chidamide was dramatically enhanced in H929 (Figure 4A, left panel, 3 vs. 4, *P* = 0.0095) and U266 (Figure 4A, right panel, 3 vs. 4, *P* = 0.0079).

As revealed by transwell invasion assay, compared with nc H929 cells, SDHA overexpression cells had a lower invasion ability (Figure 4B, left panel, *P* = 0.0412). Chidamide-treated H929 (Figure 4B, left panel, *P* = 0.0279) and U266 (Figure 3B, right panel, *P* = 0.0189) cells achieved a lower percentage of cell invasion than those treated with DMSO. Interestingly, when SDHA was overexpressed, this inhibition effect on invasive ability was more notable in H929 (Figure 4B, left panel, *P* = 0.0467) and U266 cells (Figure 4B, right panel, *P* = 0.0287). Above results revealed that SDHA overexpression was a key to inhibit proliferation and invasion and promote the effect of chidamide in MM cells.

CCK8 assay was used to determine if drugs had synergistic effect. When SDHA was overexpressed in H929 and U266 cells, the combination index (CI) of chidamide and bortezomib (Figures 4C,D, left panel) or lenalidomide (Figures 4C,D, right panel) was decreased compared with nc MM cells at the same inhibition rate (FA) level, which indicated an enhanced synergistic effect of drugs.

**FIGURE 5:**
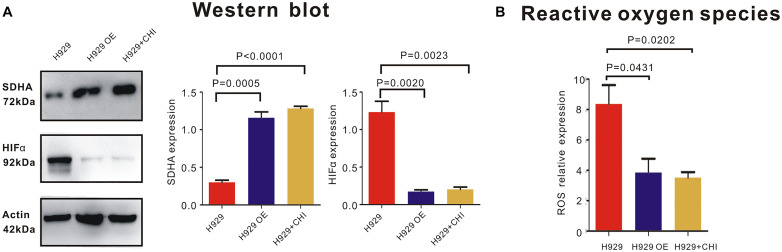
*SDHA* overexpression suppressed HIFα-ROS axis in MM cells. **(A)** Western blot showed that expression of HIFα in *SDHA* overexpression (OE) H929 cells was significantly lower than nc H929 cells. Chidamide decreased expression of HIFα in nc H929 cells. **(B)** Mitochondrial ROS production was determined by CM-H2DCFDA staining and flow cytometry. Production of ROS was decreased in SDHA overexpression (OE) H929 cells. Chidamide decreased the production of ROS.

In order to expound the effect mechanism of SDHA in MM cells, western blot was performed to show the expression of relative molecules. Here, when SDHA was overexpressed in H929 cells, HIFα was significantly downregulated (Figure 5A). Similarly, after treating with chidamide, SDHA was upregulated and HIFα was downregulated (Figure 5A). Moreover, When SDHA overexpressed in H929 cells, production of ROS was decreased significantly too (Figure 5B, *p* = 0.0431). Chidamide decreased the production of reaction oxygen species (ROS) (Figure 5B, *p* = 0.0202).

## Discussion

In our present study, we identified that SDHA was a crucial molecule in mediating the proliferation and invasion ability of MM cells, and a good prognosis factor in MM patients. We found SDHA was low expressed in MM patients, and patients with relatively high expression of SDHA had a long OS and PFS. Cell biological function analysis revealed an attenuated proliferation and invasion ability in MM cells after SDHA overexpressed. Chidamide could exert the function of anti-tumor by increasing expression of SDHA in MM.

Succinate dehydrogenase subunit A was relatively high expressed in normal volunteers, and low expressed in MM patients. We found that expression of SDHA in recurrent MM patients was lower than that in newly diagnosed patients (13). Thus, high expression of SDHA might be regarded as a good prognosis factor of MM. We tried to verify the relationship between OS or PFS and expression of SDHA. Single factor analysis showed that patients with SDHA high expression had better prognosis than other patients. Although we could draw the conclusion that high SDHA expression was correlated with better outcomes of MM patients through K-M curve in Figure 1, we could not quantify the specific survival probability of a MM patient even if we had all clinical profiles of this patient. The nomogram was constructed to solve this problem. SDHA high expression was an independent predictive factor in the stepwise Cox regression model and the hazard ratio was 0.081 compared to SDHA medium and low expression after eliminating the influence of other risk factors. Meanwhile, R-ISS staging and percentage of plasma cells in bone marrow were also involved in the prognosis prediction model. In our research, the nomogram (Figure 2A) was generated to predict 1- and 2-year overall survival (OS) probability based on the statistically significant variables in the multivariate stepwise Cox regression model. First, we could match the score of each factor in the nomogram and added them together to get the total points. Then, we drew the vertical line from the scale of total points to obtain 1- and 2-year OS probability. After the prognostic model was generated, we evaluated the agreement and discrimination of the model. The calibration curve (Figure 2B) was plotted to evaluate the agreement of our nomogram. Calibration curve reflects the agreement between observed outcomes and predictions. A calibration plot has the predicted probabilities on the *x*-axis, and the mean observed outcome on the *y*-axis, and a perfect calibration should lie on or around a 45 degree line of the plot. On the other hand, discrimination refers to the ability of a prediction model to differentiate between two outcome classes. The c-index can be interpreted as the probability that a subject with an outcome is given a higher probability of the outcome by the model than a randomly chosen subject without the outcome. A value of 0.5 indicates that the model has no discriminatory ability, and a value of 1.0 indicates that the model has perfect discrimination. In our research, the c-index was 0.871 (95% CI, 0.808–0.935).

As high expression of SDHA reflected longer OS and PFS in MM patients shown by clinical data, we then constructed SDHA steadily overexpressed MM cell lines to verify the biological effect of SDHA high expression. Deficiency of SDHA inhibited TCA cycle. MM cells activated HIFA pathway as compensation for abnormal oxygen metabolism. HIF signaling cascade is affected by hypoxia. Under the condition of hypoxia, the cells usually differentiate continuously and promote angiogenesis, which is very important for cancer cells (21). Moreover, the abnormal microenvironment results in the anomalous activation of transcription factors. Changes in the balance of growth factors, chemokines, cytokines and ROS lead tumor cells to be proliferative and invasive. HIF induces hypoxia pathway activation, which is very important for tumor growth (22).

For newly diagnosed MM patients, proteasome inhibitor bortezomib used alone or in combination with other drugs could get high remission rate (23). As a kind of immunomodulator, lenalidomide combining with low dose of dexamethasone is an effective oral therapy which commonly used in initial MM treatment (24). However, most patients would turn to relapse despite the fact that initial MM may get remission by current chemotherapy (25). So, more agents with new mechanisms should be discovered to treat MM. In our previous study, we had shown that *in vitro*, chidamide inhibited proliferation and invasion of H929 cells, and this effect vanished after knocking down SDHA. In H929 cells, lenalidomide and bortezomib had synergistic effect with chidamide to some extent, and similarly this effect was attenuated by SDHA siRNA. During this study, we added second MM cell line OPM2 into these above study and found consistent results. Also we previously showed that when SDHA was knocked down in MM cells, expression of HIFα was increased followed by increased ROS production (13). These data in turn revealed that SDHA was the key molecule which suppressed proliferation, invasion and synergistic effect of drugs. In this study, we proved high expression of SDHA inhibited biological behavior mentioned above of MM cells and enhanced the anti-tumor effect of chidamide.

Chidamide is a kind of HDACi which is developed by Chinese (26). The most important function of HDACi is enhancing expression of tumor suppressor genes and this effect has already been applied in clinical (27). At present, there have been a few studies on histone acetylation regulation of MM. The expression of HDAC in MM is up-regulated, and the up-regulated HDAC is associated with poor prognosis of MM (28). In the PANORAM clinical trial, all HDAC inhibitor panobinostat combined with bortezomib and dexamethasone was used to treat relapsed and refractory MM, and the results showed that the progression free survival of patients was significantly improved (29). Panobinostat was also launched in the United States in 2015. Abnormal histone acetylation of MM cells can also lead to tumor heterogeneity and abnormal activation of multiple conduction pathways. Application of HDAC inhibitors can downregulate the expression of cyclins, CDKs and other oncogenes, and activate all kinds of caspase and promote the apoptosis of MM cells (30). Ramakrishnan V et al. showed synergistic effect on killing MM cells with HDAC inhibitor LBH589 combined with mTORC1 inhibitor (31). J. Moreaux et al. determined gene expression profiles of five MM cell lines treated with trichostatin A (TSA) to create a histone acetylation (HA) score predicting HDACi efficacy, and patients with the highest HA Score had the shorter overall survival (32). Similarly, Gerwin Heller et al. analyzed global changes in gene expression profiles of three MM cell lines to determine molecular effects of 5-aza-2′-deoxycytidine (Aza-dC) and/or trichostatin A (TSA) in multiple myeloma (MM) and identified some new targets for epigenetic abnormalities in monoclonal gammopathies (33). Angelique Bruyer et al. reported a new gene expression-based score to predict MM cell sensitivity to the combination of DNMTi/HDACi, and treatment with DNMTi/HDACi downregulated IRF4 and MYC expression which appeared to induce a mature gene expression profile in myeloma cell lines (34). Ken Maes et al. investigated the transcriptional response after *in vivo* treatment with the HDACi quisinostat or DNMTi decitabine using the murine MM model. They found treatment of 5T33MM mice with epigenetic modulating agents led to the translation of gene signatures to predict overall survival of MM patients (35).

Chidamide has been proved effective in various kinds of malignancies. There are two main anti-tumor mechanisms of chidamide. One is directly inhibiting tumor cells (36, 37) and the other one is immunoregulation. Chidamide is able to enhance the function of NK cells and effector T lymphocytes (38), and it is effective in lung and liver cancers through inhibiting epithelial-mesenchymal transition (EMT) of tumor cells (39, 40). Now, chidamide has been already applied in relapsed and refractory PTCL and involved in conventional chemotherapy regimens (41). Previously, we have reported that chidamide was effective in inhibiting proliferation of MM cells (13).

Here, we showed the details of RNA sequencing of BMMCs from MM patients treated with chidamide or not, including GO analysis, enrichment of KEGG, and DEG analysis. Interestingly, TCA cycle regulation was ranked at the top of KEGG analysis. As the result of DEG analysis, SDHA ranked at the top of the DEG list. These results revealed that SDHA-mediated regulation of TCA cycle might play an important role in the effect of chidamide. What’s more, we proved chidamide increased H3K27 acetylation signal enrichment of histone of SDHA. According to its structure, HDACi can be divided into several categories. The first type is fatty acids which are represented by VPA. As this kind of HDACi has very low activity of inhibiting HDAC and its high metabolism degradation rate, high concentration is needed in clinical application (42). SAHA is a kind of oximate, which has been used to treat cutaneous T-cell lymphoma (43). While, chidamide belongs to benzamide. As shown in Supplementary Figure S2, only chidamide increased expression of SDHA. However, chidamide is a broad spectrum HDACi (44), so SDHA may not be the only target of chidamide in MM. In this study, we also made an estimate of different expression of another 4 DEGs in MM cells treated with or without chidamide. Although result of ChIP sequencing showed H3K27 site of SDHA was significantly acetylated by chidamide and acetylation of the other 4 DEGs was not as obvious as SDHA, DEGs such as ITGA7, MESI3, FCER2, and MRPL30 might also have important functions in chidamide treating MM. In addition, the GO and KEGG analysis also reflected a transcriptional panel of how chidamide targeting MM cells. Endothelium development was the main activated function in GO analysis, which indicated angiogenesis might be also a highlight points in MM.

**FIGURE 6:**
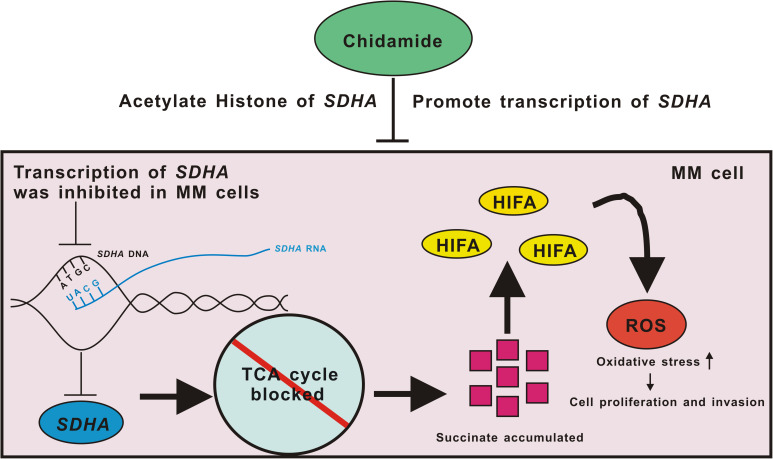
Pattern diagram of chidamide-SDHA axis in MM cells. Transcription of *SDHA* was inhibited in MM cells. Expression of SDHA was obviously decreased which led TCA cycle to be blocked and then succinate accumulated. HIFA could not be degenerated which formed pseudo-hypoxia circumstance. Increased ROS raised oxidative stress in MM cells, which promoted proliferation and invasion of tumor. Chidamide acetylated H3K27 site of *SDHA*, and promoted expression of SDHA, in order to inhibit proliferation and invasion of MM cells.

When it comes to specific mechanisms, based on the existing literature reports and our data, we propose a possible mechanism of conjecture. Deficiency of SDHA, which was targeted by chidamide, led TCA cycle to be blocked and succinate accumulated. This pseudoanoxic microenvironment stimulated a high expression of HIFα and increased production of ROS. MM cells became highly proliferative and invasive eventually (Figure 6). We are now trying to find the deep mechanisms between SDHA and MM. In this study, we found that transcription of SDHA was inhibited, and expression of SDHA remained at a low level in MM cells. High expression of SDHA may become a new positive prognostic factor of MM patients and chidamide can inhibit MM cells by enhancing expression of SDHA. It may become a new strategy to treat MM in the future.

## Data Availability Statement

The datasets presented in this study can be found in online repositories. The names of the repository/repositories and accession number(s) can be found below: NCBI Gene Expression Omnibus (GSE156502).

## Ethics Statement

The studies involving human participants were reviewed and approved by the Review Board of Zhongshan Hospital, Fudan University (Shanghai, China). The patients/participants provided their written informed consent to participate in this study.

## Author Contributions

YS and PL designed the experiments. YS, JJ, and JX performed the experiments. YS and ZX analyzed the data. YS, ZX, and PL provided professional writing services and materials and wrote the manuscript. All authors met the criteria for authorship and approved the final manuscript for publication.

## Conflict of Interest

The authors declare that the research was conducted in the absence of any commercial or financial relationships that could be construed as a potential conflict of interest.
