# Supplementary material for: High Expression of Succinate Dehydrogenase Subunit A Which Is Regulated by Histone Acetylation, Acts as a Good Prognostic Factor of Multiple Myeloma Patients
Source: Front Oncol. 2020 Sep 10;10:563666. doi: 10.3389/fonc.2020.563666 (PMC7511799; doi:10.3389/fonc.2020.563666)
Supplement: Supplementary file 1 [file Data_Sheet_1.PDF]

Supplementary Material

1 Supplementary Figures and Tables

1.1 Supplementary Figures

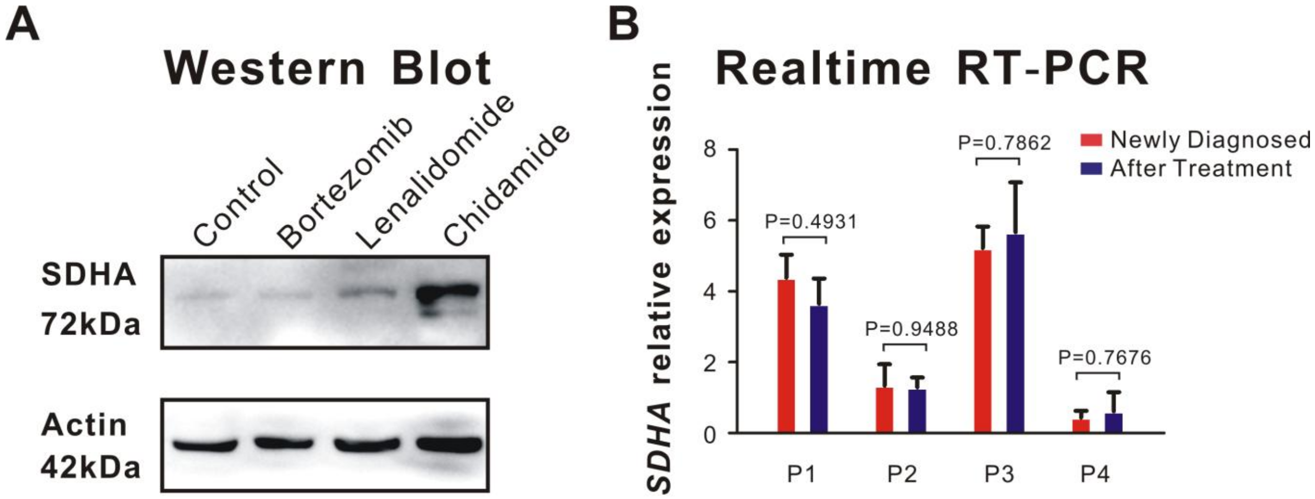

Supplementary Figure 1. Expression of *SDHA* remained steadily after treatment.

(A) Expression of protein was determined by western blot. H929 cells were treated by bortezomib (10.67μM), lenalidomide (2.48mM) or chidamide (6μM) respectively. Compared with H929 cells treated with isometric DMSO, expression of SDHA in cells treated with bortezomib and lenalidomide had no significant difference. Expression of SDHA was increased only in chidamide-treated cells. **(B)** BMMCs of Four MM patients were randomly selected. Realtime RT-PCR analysis showed that *SDHA* expression had no significant difference between BMMCs extracted at newly diagnosed and BMMCs extracted after treatment. P1-P4 patients underwent VTD, VCD, VCD and RD chemotherapy regimens respectively. V=bortezomib, T=thalidomide, C=cyclophosphamide, D=dexamethasone, R=lenalidomide.

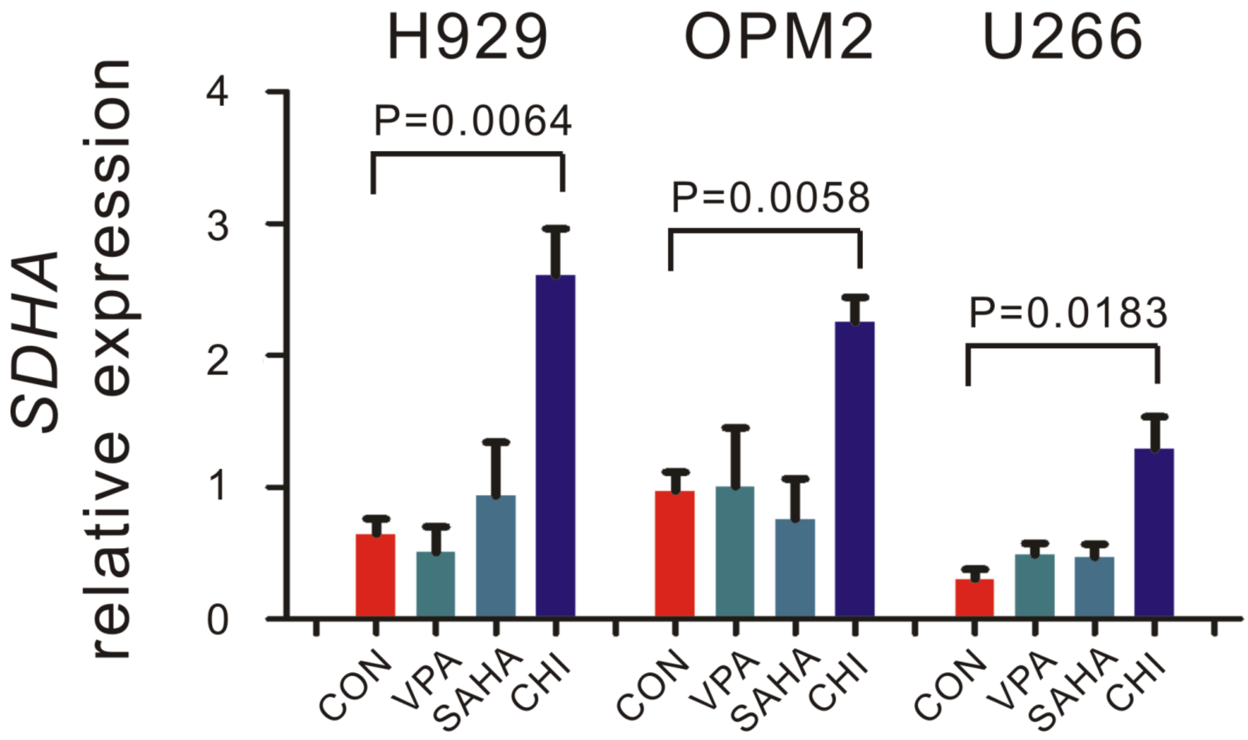

Supplementary Figure 2. Effect of three HDACi on expression of *SDHA*.

MM cells (H929, OPM2 and U266) were treated by valproic acid (VPA, 2mM), vorinostat (SAHA, 10μM) and chidamide (6μM) respectively. Compared with cells treated with isometric DMSO, expression of SDHA in cells treated with VPA and SAHA had no significant difference. Expression of SDHA was increased only in chidamide-treated cells.

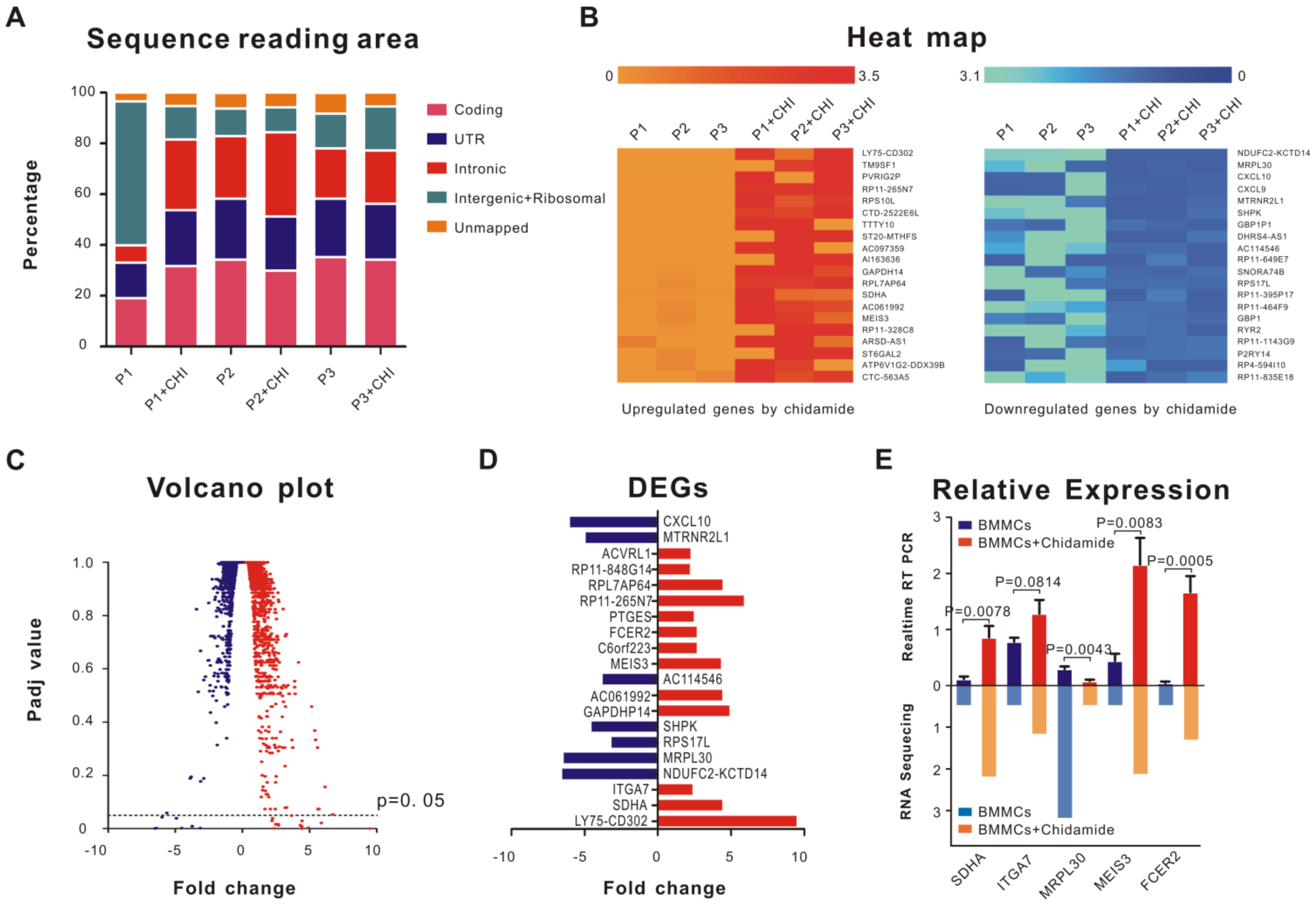

Supplementary Figure 3. Basic facts of RNA sequencing.

BMMCs were isolated by Ficoll centrifugation from three patients and then cultured with 6μM chidamide or isometric DMSO for 24 hours. RNA was extracted from BMMCs by trizol method. **(A)** Distribution of sequence reading area of total six samples. **(B)** Heat maps of partial genes in RNA sequencing. Left panel showed genes upregulated by chidamide and genes downregulated by chidamide was showed in right panel. **(C)** Volcano plots of all genes involved in RNA sequencing. Genes were plotted as blue (downregulated genes after chidamide treatment) and red (upregulated genes after chidamide treatment) points. **(D)** The top 20 DEGs which had the lowest 20 padj value, fold change>2 and FDR<0.05 were showed in this panel. Red columns represented expression of upregulated genes after treated by chidamide and blue columns represented expression of downregulated genes after treated by chidamide. **(E)** 5 genes were chosen from Supplementary Table 2. Non-coding genes were excluded and top 5 genes which were relevant to malignancies were chosen by previous references. BMMCs were isolated by Ficoll centrifugation from five patients and then cultured with 6μM chidamide or isometric DMSO for 24 hours. *SDHA*, *MEIS3* and *FCER2* were significantly upregulated by 6μM chidamide; MRPL30 was significantly downregulated by 6μM chidamide.

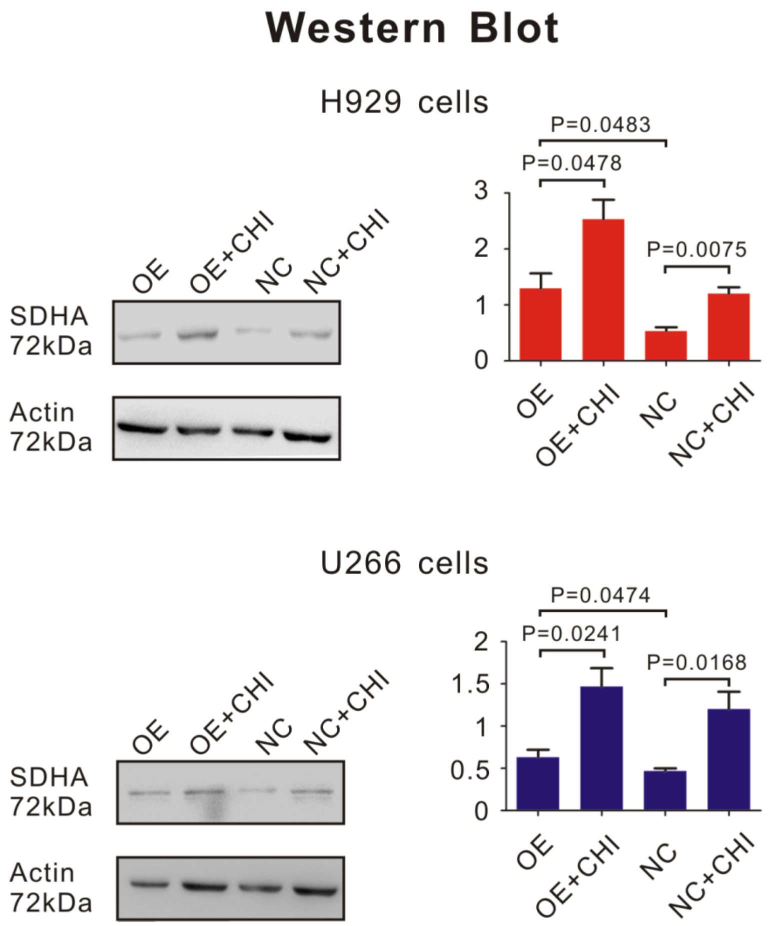

Supplementary Figure 4. the SDHA levels of after SDHA overexpression (OE), treatment with chidamide (CHI) and both (OE+CHI).

SDHA overexpression H929 and U266 cells were constructed by lenti-virus transfection. Expression of SDHA in OE cells was significantly higher than NC cells (p=0.0483 and 0.0474 in H929 and U266 cells respectively). Expression of SDHA in chidamide treated cells was significantly higher than DMSO treated cells (p=0.0075 and 0.0168 in H929 and U266 cells respectively). Expression of SDHA in chidamide treated OE cells was significantly higher than DMSO treated OE cells (p=0.0478 and 0.0241 in H929 and U266 cells respectively).

1.2 Supplementary Tables

Variables	P value	HR
Middle SDHA expression	0.1004	0.374
High SDHA expression	0.0235	0.081
R-ISS II	0.7708	960
R-ISS III	0.7529	1669
Percentage of plasma cells in bone marrow	0.0201	1.038

Supplementary Table1. Cox multivariate regression analysis of variables.

**Genes**	**Functions**	**Expression**
LY75-CD302	The sequence between CD75 and CD302. CD302 participates in cell adhesion and migration. CD302 negatively regulates angiogenesis.	Upregulation
SDHA	Succinate dehydrogenase subunit A,SDHA was a key enzyme of TCA cycle, and regulates production of ROS.	Upregulation
ITGA7	ITGA7 mediates integration of cell-cell and cell-cell matrix and involved in cell migration, differentiation and invasion.	Upregulation
NDUFC2-KCTD14	The sequence between NDUFC2 and KCTD14. The function is not clear.	Downregulation
MRPL30	A kind of mitochondrial ribosomal protein.	Downregulation
RPS17L	A kind of ribosomal protein.	Downreglation
SHPK	Sedoheptulokinase assists glucose to transported into cells.	Downregulation
GAPDHP14	A pseudogene and have no reported function.	Upregulation
AC061992	Have no reported function.	Upregulation
AC114546	Have no reported function.	Downregulation
MEIS3	A kind of transcriptional regulatory factor, and it is associated with development of pulmonary epithelial cells	Upregulation

	and midbrain.	
C6orf223	Have no reported function.	Upregulation
FCER2	FC fragment of IgE receptor 2, and it is associated with proliferation and differentiation.	Upregulation
PTGES	Prostaglandin E synthelase was induced by IL-1B and TP53.	Upregulation
RP11-265N7	Have no reported function.	Upregulation
RPL7AP64	Have no reported function.	Upregulation
RP11-848G14	Have no reported function.	Upregulation
ACVRL1	A receptor involved in TGFβ pathway and it is associated with angiogenesis in tumors.	Upregulation
MTRNR2L1	A pseudogene and have no reported function.	Downregulation
CXCL10	CXCL10 binds to CXCL3 to activate monocyte and promote migration of NK cells and T lymphocytes. It can also regulate expression of some adhesion molecules.	Downregulation

Supplementary Table2. The detailed lists of the top 20 DEGs.

NO.	GO	Upregulated genes	P value	Downregulated genes	P value
1	BP	Blood vessel maturation	4.226e-06	Organonitrogen compound	3.644e-05
2	BP	Positive regulation of endothelial cell	1.583e-05	Ribose phosphate metabolic process	2.978e-04
3	BP	Regulation of endothelial cell differentiation	5.682e-05	Nucleotide metabolic process	5.073e-04
4	BP	Anatomical structure maturation	0.0001689	Nucleoside phosphate metabolic process	5.265e-04
5	BP	Positive regulation of epithelial cell differentiation	0.0002061	Cellular response to interleukin-13	5.997e-04
6	BP	Endothelial cell differentiation	0.0005815	Negative regulation of myoblast fusion	5.997e-04
7	BP	Endothelium development	0.0008865	Nucleobase-containing small molecule metabolic process	6.602e-04
8	BP	Adherens junction organization	0.001019	Cellular response to lipopolysaccharide	7.486e-04
9	BP	Regulation of epithelial cell differentiation	0.001036	Cellular response to molecule of bacterial origin	8.110e-04
10	BP	L-serine catabolic process	0.001259	Negative regulation of syncytium formation by plasma membrane fusion	8.995e-04

1	CC	Cell surface	9.045e-05	Ribosomal subunit	1.158e-03
2	CC	Nuclear envelope lumen	0.003118	Ribosome	2.292e-03
3	CC	External side of plasma membrane	0.00335	Mitochondrial inner membrane	9.736e-03
1	MF	L-threonine ammonia-lyase activity	0.0008583	Sedoheptulokinase activity	3.066e-04
2	MF	L-serine ammonia-lyase activity	0.001287	CXCR3 chemokine receptor binding	1.532e-03
3	MF	Prostaglandin-E synthase activity	0.001287	Structural constituent of ribosome	1.609e-03
4	MF	Transforming growth factor beta receptor activity, type I	0.001716	cAMP-dependent protein kinase regulator activity	2.144e-03
5	MF	Activin receptor activity, type I	0.001716	CXCR chemokine receptor binding	5.202e-03
6	MF	Ammonia-lyase activity	0.002144		
7	MF	IgE binding	0.002144		
8	MF	BMP receptor activity	0.002144		
9	MF	Activin-activated receptor activity	0.003001		
10	MF	Transforming growth factor beta-activated receptor activity	0.003857		
1	KEGG	Citrate cycle (TCA	0.02725	Cytosolic DNA-	1.851e-02

		cycle)		sensing pathway	
2	KEGG	Cysteine and methionine metabolism	0.03062	RIG-I-like receptor signaling pathway	2.430e-02
3	KEGG	Arachidonic acid metabolism	0.04895	Ribosome	3.007e-02
4	KEGG			Toll-like receptor signaling pathway	3.481e-02

Supplementary Table3. The top 10 significantly enriched GO and KEGG terms.
